# Incipient primary biliary cirrhosis/autoimmune hepatitis overlap or hepatitic form of primary biliary cirrhosis: a case report

**DOI:** 10.1186/1757-1626-2-7491

**Published:** 2009-05-27

**Authors:** Ranjana W Minz, Seema Chhabra, Ritu Aggarwal, Ashim Das, Biman Saikia, Yogesh K Chawla

**Affiliations:** 1 Department of Immunopathology, Postgraduate Institute of Medical Education & Research,Sector-12, Chandigarh - 160 012,India; 2 Department of Hepatology, Postgraduate Institute of Medical Education & Research,Sector-12, Chandigarh - 160 012,India

## Abstract

A 42 year old asymptomatic female detected as incipient Primary Biliary Cirrhosis/Autoimmune Hepatitis overlap during routine checkup. The biochemical profile showed evolution from a mildly deranged liver function test in 2004 along with increased erythrocyte sedimentation rate to a 4 times elevation of alkaline phosphatase in 2006 with mildly deranged alanine transaminase. Autoimmune markers demonstrable were Anti mitochondrial antibody M_2_ and sp100. Histopathology showed dual features, dominant findings were of autoimmune heptatitis. Features consistent with Primary Biliary Cirrhosis were minimal with an occasional portal tract showing paucity of bile ducts and occasional bile duct proliferation. Human leucocyte antigen DR/DQ genotype was as follows: DRB1*03, DRB1*07, DQB1*02, DQB1*04.

## Introduction

Autoimmune diseases of liver are defined relatively simply by clinical, biochemical, radiologic and histopathological criteria into those characterized predominantly by hepatitis (AIH) or by cholestasis (PBC) [1]. PBC & AIH are the two main immune mediated liver diseases that lie at opposite ends of the spectrum of autoimmune liver diseases and differ remarkably with respect to pathological findings and therapeutic intervention. Some patients present with features of autoimmune liver disease that do not conform uniquely to any of the established criteria for the diagnosis of autoimmune hepatitis (AIH) or primary biliary cirrhosis (PBC). Various names, mostly overlap syndrome, have been used to describe these cases. We report a unique case of an asymptomatic 42-yr-old female detected as incipient PBC/AIH overlap during routine annual checkup.

## Case presentation

A 42-yr-old Indian female, G2P2 with 2 healthy sons, residing in Cyprus, was detected to have a raised ESR during a routine health check-up in 2000. This was the only lab investigation found abnormal in the subsequent annual check-ups. She is a non alcoholic, non smoker with no significant medication history or any history of blood transfusion. In 2004, again during the annual heath check-up, it was detected that in addition to an increased ESR (33 with normal range of 0-20, London Clinic), she had raised liver enzymes with ALT: 64 U/L (5-65), GGT:100 U/L (10-60) and alkaline phosphatase: 169 U/L (40-165). In 2005, an ultrasound of abdomen and MRCP were also performed during the annual check up. USG findings showed contracted gall bladder and acoustic shadows due to non-functioning gall bladder with a small stone. No focal lesion was seen in the liver. There was mild enlargement of the right lobe of the liver. The pancreas, kidneys and spleen were normal. Magnetic resonance cholangio-pancreatography (MRCP) showed filling defects in gall bladder due to gall stones. The bile ducts and pancreas were normal. All the liver enzymes were tested thrice in 2005 in February, April and September and at all occasions found to be significantly elevated. Patient underwent cholecystectomy in October, 2005. Peroperative liver biopsy was performed that showed only mild fatty degeneration. In 2006, the liver enzymes remained elevated, the levels were as follows: alkaline phosphatase: 244 U/L, ALT: 79 U/L, GGT: 157 U/L. Patient underwent screening for hepatitis E, A, B & C and autoimmune markers. All the viral markers were found negative. Antinuclear antibodies were positive on autoimmune serology. Liver biopsy was reviewed again.

**Figure 1.**
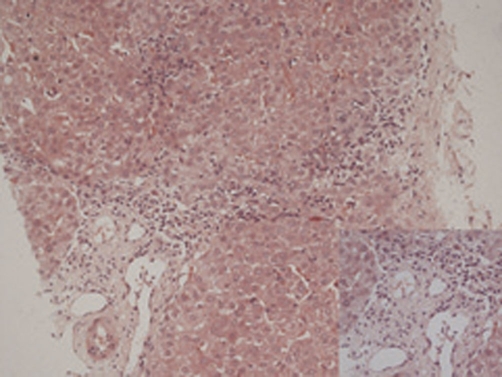
Photomicrograph of liver biopsy showing lympho-plasmacytic infiltrate and paucity of bile ducts in portal triad, and foci of lobular inflammation (H&E, x200). Inset showing high power view of the portal triad (H&E, x400).

**Figure 2.**
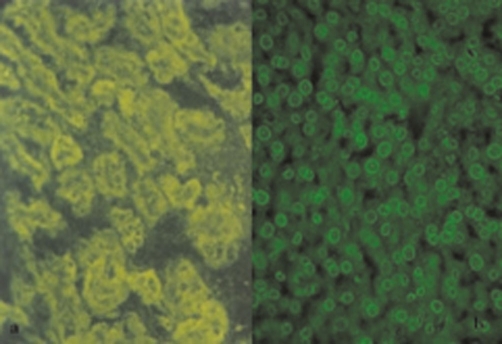
**(a)** Indirect immunofluorescence photomicrograph demonstrating strong AMA positivity on rodent kidney tissue (x200); **(b)** Indirect immunofluorescence photomicrograph demonstrating focal rim pattern with nuclear dots on composite rodent tissue (x400).

**Figure 3.**
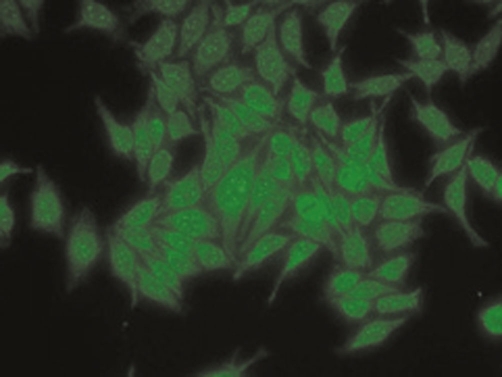
Indirect immunofluorescence photomicrograph of Hep2 cell lines showing typical multiple nuclear dots (MNDs) pattern (x200).

**Figure 4.**
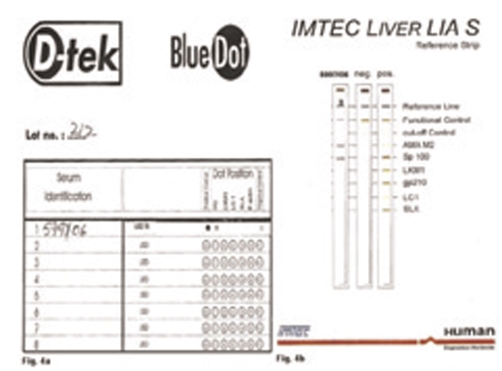
**(a)** Immunoblot with 5 antigens showing positivity for AMA M_2_; **(b)** Immunoblot with 6 antigens showing positivity for AMA M_2_ and sp100.

The liver function tests were performed in 2006 at our institute and the results were as follows: AST: 53 U/L (3-39), ALT: 68 U/L (2-40), total bilirubin: 0.6 mg/dl (0.2-0.8 mg/dl), alkaline phosphatase: 640 U/L (98-306). Serum iron studies and α1-antitrypsin levels were within normal limits. Liver biopsy was twice reviewed by expert histopathologists and showed maintained lobular architecture with a moderate chronic inflammatory cell infiltrate including some plasma cells in the portal tracts. There was a focal periportal extension of inflammatory cells into the adjoining hepatocytes. Some portal tracts showed neutrophilic infiltrate involving the bile duct epithelium. The portal tracts showed mild to moderate fibrosis with focal porto-portal bridging fibrosis. Several foci of lobular inflammation were seen with a few acidophilic and preacidophilic bodies. There was mild Kupffer cell hyperplasia and sinusoidal lymphocytic infiltration. (Figure 1) Few portal tracts were devoid of bile ducts but focal ductular proliferation was noted in some. In view of these features, a diagnosis of autoimmune hepatitis with a differential diagnosis of AIH/PBC overlap was considered in this case. The patient was thoroughly worked for autoimmune markers in the Department of Immunopathology. The presence of antinuclear antibodies (ANA), anti smooth muscle antibodies (ASMA), antimitochondrial antibodies (AMA) and liver-kidney microsomal antibodies (LKM) was determined using indirect immunofluorescence on a composite of rat liver tissues (liver, kidney and stomach). Patient was found to be AMA positive (++++) and ANA (+++, rim pattern) positive in 1:80 dilution (Figure 2a and 2b). ANA pattern was further analyzed using Hep-2 cell lines that showed ++ to +++ peripheral pattern and multiple nuclear dots (MND) (Figure 3). AMA-M2 ELISA was performed in triplicate; the mean optical density was 0.769/cut-off 0.388. D-tek Liver5 DOT simultaneously testing for AMA-M2, LKM-1, LC-1, SLA & F-actin was done, that again showed positivity for AMA-M2 (Figure 4a). On further testing on the Imtech LIVER LIA S blot (Human GmBH), AMA-M2 and nuclear body protein sp100 were demonstrable, whereas gp210 was negative (Figure 4b). Thus serology favored a diagnosis of primary biliary cirrhosis. To confirm the genetic susceptibility for autoimmunity, HLA DNA testing was carried out that showed a genotype of DRB1*03, DRB1*07, DQB1*02, DQB1*04. So based on biochemical, immunological and histopathological findings and genotype profiling, a diagnosis of incipient PBC/AIH overlap was offered in this case. To look for concomitant autoimmune disease, testing for thyroid microsomal antibodies was also done and that was negative. Patient also had gestational diabetes in last trimester of her first pregnancy in 1994. Since then blood sugar levels have remained within normal limits. Patient's mother is diabetic and also has mild arthritis.

Since September 2006 patient is taking Urosofalk. After 2 years of follow-up, she shows biochemical improvement with serologic profile remaining more or less stable on indirect immunofluorescence screening; however ELISA and immunoblot record a quantitative decline in AMA-M2 positivity.

## Discussion

PBC was first described in India from our centre in 1973 as a rare entity [1]. The largest series from India describes late detection of PBC and PBC/AIH overlap (26%) due to low intensity of screening and evaluation of liver function [2].

There is currently no consensus on what constitutes an autoimmune overlap syndrome. In true overlap syndrome, the patient has clinical, serologic and histologic characteristics of two conditions either at the same time or during the course of their illness [3]. In a study by Amrapurkar et al [4] all 6 cases of PBC/AIH overlap were detected in a late stage i.e cirrhotics and in a case report from Pune [5], the female patient presented with decompensated cirrhosis conforming to the findings of Sarin et al [2]. This index case has been detected in the incipient stage, where by using stringent diagnostic criteria (International Autoimmune Hepatitis criteria [6]: 7/15; recent simplified criteria for AIH [7]: 5/7; PBC criteria: 3/3) the patient may be labeled as a hepatitic variant of PBC. However, the predominant features of AIH on histopathology are out of place in a case of PBC. Taking the presence of DR3 in this patient, it is tempting to agree with Lohse's observation that a majority of patients with overlap syndrome have the characteristic HLA haplotype of AIH namely HLA-B8, DR3 or DR4 [8]. HLA DR3 is a well known Caucasoid susceptibility gene for type 1 AIH and its presence in our patient dictates the nature of her disease. Patients with HLA-DR3 haplotype present at an early age and enter remission less frequently during therapy.

The liver biopsy is invaluable in making a diagnosis of co-existent liver disease [9]. The index case highlights the importance of liver biopsy in making a diagnosis of PBC/AIH overlap.

Care must be taken while assigning AMA positive status, because AMA may not be detected by typical immunofluorescence, but may be detectable by other means such as ELISA or immunoblot using the recombinant PDC-E2 target autoantigen of M2 antibodies. Our patient was thoroughly worked up by all 3 means and finally diagnosed as AMA M2 positive.

Antinuclear antibodies are also detectable in approximately 50% of subjects with PBC. On indirect immunofluorescence on Hep2 cells to detect ANA, two labeling patterns that predominate in PBC are punctate nuclear rim and multiple nuclear dots (MND). These patterns recognize nuclear pore membrane protein gp120 and nuclear body protein sp100 respectively. These ANAs are highly specific for PBC and detected in approximately 25% of patients [10]. Our patient showed 5-8 nuclear dots on indirect immunofluorescence and positivity for sp100 by immunoblotting but was negative for gp120 thus providing another serologic evidence for PBC.

The recognition of autoimmune overlap syndrome is not only important from a classification standpoint, but it may have implications for therapy as recent studies demonstrate that PBC/AIH overlap has similar outcome as AIH [9]. A combination of ursodeoxycholic acid and corticosteroids is required in most patients to obtain a complete clinical and biochemical response.

It is also emphasized that there is need for closely following each and every patient of autoimmune liver disease with detailed serological profiling, histopathology of liver biopsy and HLA DNA DR/DQ analysis to unravel and comprehend the true PBC/AIH overlap and distinguish it from hepatitic form of PBC and AIH. Such an approach will allow detection of the bulk of autoimmune diseases of the liver in India at as incipient/early a stage as ours, when therapy will be highly beneficial.

## Conclusion

This case is presented to highlight that a thorough serological, biochemical and histopathological workup along with DNA DR/DQ profiling is essential to diagnose PBC/AIH overlap which is hypothesized to be in incipient stage.

## Abbreviations

AIH: Autoimmune hepatitis
ALT: Alanine aminotransferase
AMA: Antimitochondrial antibodies
ANA: Antinuclear antibodies
ASMA: Anti smooth muscle antibodies
AST: Aspartate aminotransferase
ELISA: Enzyme-Linked Immunosorbent Assay
ESR: erythrocyte sedimentation rate
HLA DNA: Human leukocyte antigen-Deoxyribonucleic acid
LKM: Liver-kidney microsomal antibodies
MRCP: Magnetic resonance cholangio-pancreatography
PBC: Primary biliary cirrhosis
